# Identification of Intracranial Germ Cell Tumors Based on Facial Photos: Exploratory Study on the Use of Deep Learning for Software Development

**DOI:** 10.2196/58760

**Published:** 2025-01-30

**Authors:** Yanong Li, Yixuan He, Yawei Liu, Bingchen Wang, Bo Li, Xiaoguang Qiu

**Affiliations:** 1 Department of Radiation Oncology Beijing Tiantan Hospital Capital Medical University Beijing China; 2 Department of Pediatric Department Beijing Tiantan Hospital Capital Medical University Beijing China; 3 School of Basic Medical Sciences Capital Medical University Beijing China; 4 Beijing Luhe Hospital Affiliated to Capital Medical University Beijing China; 5 School of Computer and Artificial Intelligence Zhengzhou University Zhengzhou China; 6 Beijing Neurosurgical Institute Beijing China

**Keywords:** deep learning, facial recognition, intracranial germ cell tumors, endocrine indicators, software development, artificial intelligence, machine learning models, software engineering, neural networks, algorithms, cohort studies

## Abstract

**Background:**

Primary intracranial germ cell tumors (iGCTs) are highly malignant brain tumors that predominantly occur in children and adolescents, with an incidence rate ranking third among primary brain tumors in East Asia (8%-15%). Due to their insidious onset and impact on critical functional areas of the brain, these tumors often result in irreversible abnormalities in growth and development, as well as cognitive and motor impairments in affected children. Therefore, early diagnosis through advanced screening techniques is vital for improving patient outcomes and quality of life.

**Objective:**

This study aimed to investigate the application of facial recognition technology in the early detection of iGCTs in children and adolescents. Early diagnosis through advanced screening techniques is vital for improving patient outcomes and quality of life.

**Methods:**

A multicenter, phased approach was adopted for the development and validation of a deep learning model, GVisageNet, dedicated to the screening of midline brain tumors from normal controls (NCs) and iGCTs from other midline brain tumors. The study comprised the collection and division of datasets into training (n=847, iGCTs=358, NCs=300, other midline brain tumors=189) and testing (n=212, iGCTs=79, NCs=70, other midline brain tumors=63), with an additional independent validation dataset (n=336, iGCTs=130, NCs=100, other midline brain tumors=106) sourced from 4 medical institutions. A regression model using clinically relevant, statistically significant data was developed and combined with GVisageNet outputs to create a hybrid model. This integration sought to assess the incremental value of clinical data. The model’s predictive mechanisms were explored through correlation analyses with endocrine indicators and stratified evaluations based on the degree of hypothalamic-pituitary-target axis damage. Performance metrics included area under the curve (AUC), accuracy, sensitivity, and specificity.

**Results:**

On the independent validation dataset, GVisageNet achieved an AUC of 0.938 (*P*<.01) in distinguishing midline brain tumors from NCs. Further, GVisageNet demonstrated significant diagnostic capability in distinguishing iGCTs from the other midline brain tumors, achieving an AUC of 0.739, which is superior to the regression model alone (AUC=0.632, *P*<.001) but less than the hybrid model (AUC=0.789, *P*=.04). Significant correlations were found between the GVisageNet’s outputs and 7 endocrine indicators. Performance varied with hypothalamic-pituitary-target axis damage, indicating a further understanding of the working mechanism of GVisageNet.

**Conclusions:**

GVisageNet, capable of high accuracy both independently and with clinical data, shows substantial potential for early iGCTs detection, highlighting the importance of combining deep learning with clinical insights for personalized health care.

## Introduction

Intracranial germ cell tumors (iGCTs) are primary malignant brain tumors of the central nervous system, predominantly affecting children and adolescents [1]. In East Asian countries, iGCTs account for 15% of all pediatric and adolescent brain tumors, making them the third most common primary brain tumor [2]. In other regions, the incidence of iGCTs is only 2%-3%, classifying them as rare tumors [1]. IGCTs typically occur in the midline structures of the brain, with approximately 90% located in the pituitary and pineal regions, and a minority in the fourth ventricle and brain parenchyma [3]. IGCTs can be treated with radiotherapy and chemotherapy, with only about 20% of patients requiring surgical resection [4,5]. The symptoms of iGCTs vary depending on the tumor’s location, size, and cerebrospinal fluid dissemination. Common symptoms include headaches, nausea, vomiting, visual changes, endocrine disorders, ataxia, seizures, behavioral and cognitive changes, and cranial nerve dysfunction [6]. The manifestation and severity of these symptoms differ among individuals and according to the specific tumor characteristics. Patients often present with various symptoms and are diagnosed with an intracranial tumor through computed tomography or magnetic resonance imaging scans. However, due to the rarity of iGCTs in most regions, patients are frequently misdiagnosed with craniopharyngiomas, pituitary tumors, gliomas, or pinealocytomas and undergo unnecessary surgical resection [7]. Postoperative pathology often reveals iGCTs, necessitating subsequent radiotherapy and chemotherapy, thus indirectly leading to overtreatment [8].

According to the clinical consensus, iGCTs can be clinically diagnosed when tumor markers (eg, human chorionic gonadotropin [HCG] and alpha-fetoprotein) are elevated [7]. However, due to a lack of diagnostic experience, these tumor markers are often overlooked at initial diagnosis, leading to unwarranted surgical treatment. Therefore, developing a noninvasive, efficient, and universally applicable screening tool is crucial to provide clinicians with indicative information at the first patient visit, guiding targeted examinations. Since iGCTs patients exhibit facial feature changes compared with normal children, we hypothesize that facial recognition algorithms can be developed to alert clinicians at the initial consultation, providing personalized diagnostic approaches [9,10].

The application of facial recognition algorithms is extensive, especially those based on machine learning algorithms, which have been profoundly studied in the medical field. For instance, Bottinelli et al [11] used a binocular rivalry task to assess emotional processing in patients with panic disorder (PD), finding that with PD had a greater initial threat bias toward fearful faces compared with controls, indicating that threat-evoking stimuli more rapidly captured their attention. Yeung et al [12] analyzed 148 studies on facial emotion recognition in autism spectrum disorder, revealing significant impairments in recognizing all basic facial emotions in individuals with autism spectrum disorder compared with typically developing controls. Nigam et al [13] examined facial emotion recognition deficits in remitted patients with bipolar disorder and their first-degree relatives compared with healthy controls, finding significant deficits in recognizing emotions such as fear, anger, surprise, and happiness in patients with bipolar disorder compared with first-degree relatives and healthy controls. iGCTs are a type of intracranial tumor often accompanied by significant endocrine abnormalities, and facial recognition technology holds the potential for uncovering diagnostic information. Nevertheless, current research on this aspect remains unclear

Therefore, this study aims to design a deep learning-driven binary facial recognition model using frontal facial photographs of patients to first distinguish between midline brain tumors and normal controls and then differentiate iGCTs from other midline brain tumors. We will initially screen for the optimal model among commonly used deep learning (DL) models in facial recognition, using development and independent external validation datasets composed of open-source databases (Wild Young Labeled Faces) and multicenter clinical patient facial photographs. Finally, we will develop visualization software to demonstrate the practical clinical application of our model.

## Methods

### Study Design

**Figure 1:**
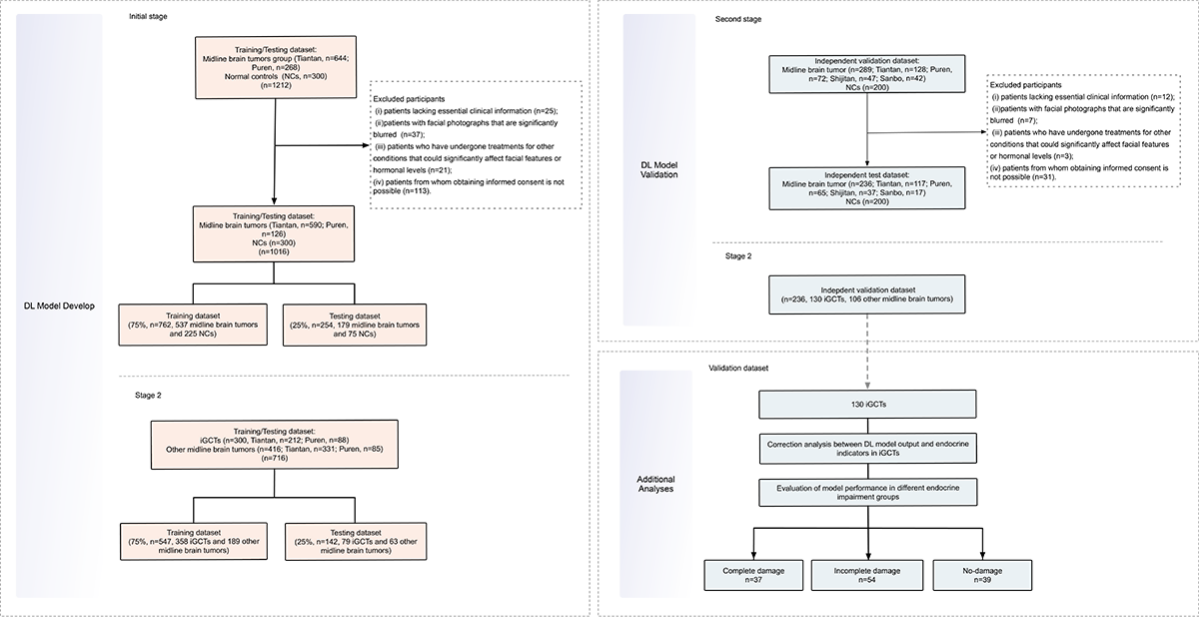
The study design and participant flow. DL: deep learning; iGCT: intracranial germ cell tumor; NC: normal control.

The objective of this multicenter retrospective diagnostic study was to develop and validate a 2 stage DL facial recognition classification model, GVisageNet, using frontal facial photographs to distinguish patients with midline brain tumors and normal controls (NCs), and further distinguish iGCTs from other patients with midline brain tumors, thereby facilitating early detection. Data collection occurred across 4 Chinese medical institutes from January 2010 to May 2023. The study design and participant flow are illustrated in Figure 1.

### Participants

Inclusion criteria were (1) patients diagnosed pathologically with midline brain tumors (specify tumors originating in the sellar region, pineal region, or the fourth ventricle); (2) availability of frontal facial photographs taken within 2 weeks before the date of diagnosis; (3) gender- and age-matched NCs from the “Wild Young Labeled Faces” dataset with age and gender matching from the GitHub repository managed by the Institute of Systems and Robotics at the University of Coimbra. Exclusion criteria were (1) patients lacking essential clinical information, including but not limited to demographics (eg, age and gender), symptoms, and hormonal levels; (2) patients with facial photographs that are significantly blurred, to the extent that key facial landmarks or features are indistinguishable; (3) patients who have undergone treatments for other conditions that could significantly affect facial features or hormonal levels; and (4) patients from whom obtaining informed consent is not possible. Finally, patients were categorized into the midline brain tumors group and NCs, and further divided into iGCTs and the other midline brain tumor groups.

### Study Setting

This investigation was systematically conducted in 3 phases, using a multicenter approach for patient recruitment and data collection. During the initial phase, NCs and patients diagnosed with midline brain tumors (including iGCTs and other midline brain tumors) were recruited from the wild young labeled faces dataset, as well as from Tiantan, Puren Hospital, Capital Medical University. This can be deleted from the text. In this phase, our goal was to construct a binary tandem DL model (GVisageNet). We randomly divided the NCs and patients with midline brain tumors (n=1016) into 2 groups, 75% for training the GvisageNet and 25% for testing its performance (762:254). Furthermore, an independent external dataset was compiled from 4 distinct medical institutions (Tiantan, Puren, Shijitan, and Sanbo Hospital, Capital Medical University) between January 2020 and May 2023, aiming to establish an independent validation dataset (n=436) for validating the DL model. Subsequently, the patients with midline brain tumors (n=716) from the first phase were randomly divided into a training dataset and a testing dataset in a 75:25 ratio to differentiate iGCTs from other midline brain tumor patients (574:142). Finally, the facial photographs of patients with midline brain tumors (n=236) from the independent external validation dataset were used as a separate validation dataset in the second phase.

Since both iGCTs and other midline brain tumors affect the brain's midline functional areas in clinical diagnosis, leading to similar facial characteristics caused by impacts on the patient’s endocrine function, differential diagnosis becomes even more challenging. However, incorrect predictions could result in misdiagnosis, misinterpretation, and inefficient use of resources. To further enhance predictive accuracy, the subsequent phase of the study aimed to develop a regression model using clinical variables marked by statistical disparities between the 2 groups (iGCTs vs other midline brain tumors). This included demographic characteristics, clinical manifestations, and hormonal profiles, where significant differences were observed. This model was then integrated with the outputs generated by GVisageNet (from the second stage), enabling the construction of an integrated hybrid model. The primary goal of this integration was to clarify the contribution of clinical data in improving the model’s predictive performance.

In the final phase of the study, the correlation analysis between the outputs of GVisageNet and 15 endocrine indicators in the independent validation dataset was conducted to explore the potential mechanism of GVisageNet. Finally, stratified studies were carried out in the independent validation dataset based on the degree of hypothalamic-pituitary-target (HPT) axis damage (complete damage, incomplete damage, and no damage), further verifying that endocrine changes are the potential mechanism behind the effectiveness of GVisageNet.

### Data Collection

Frontal views of each patient were routinely captured at the initial hospital visit using a standardized protocol. This involved a digital camera with a resolution exceeding 10 million pixels (the criteria and details of photograph taking are presented in Method S1 in Multimedia Appendix 1).

In studying the correlation between facial recognition features and endocrine functions, the selection of specific clinical indicators is designed to comprehensively assess patients’ endocrine conditions and their potential impacts on facial characteristics. This includes baseline data such as gender, age, BMI, initial symptoms, disease duration, and the primary site of the tumor, which provide an initial understanding of the patient's overall health and endocrine function. Key endocrine indicators include the adrenal axis (Adrenocorticotropic hormone), thyroid axis (thyroid stimulating hormone [TSH], triiodothyronine, free triiodothyronine [FT3], thyroxine, and free thyroxine [FT4]), growth hormone-insulin-like growth factor-I axis (growth hormone [GH], insulin-like growth factor 1, insulin-like growth factor binding protein 3 [IGFBP-3]), and gonadal axis (follicle-stimulating hormone [FSH], luteinizing hormone [LH], testosterone, estradiol, progesterone, and prolactin). Variations in these hormone levels can lead to significant changes in facial features, such as puffiness, growth retardation, or precocious puberty. In addition, routine monitoring of 24-hour urine volume, specific gravity, and osmolality indirectly reflects the patient's endocrine health status, thereby supporting the potential application of facial recognition technology in clinical diagnostics. This comprehensive data collection is crucial for exploring the link between facial characteristics and endocrine functions, holding significant academic value.

### Pathological Diagnosis

The criterion for correct GVisageNet prediction is consistency with pathological results. All patients enrolled in the study were classified according to the 2021 WHO (World Health Organization) pathological classification [14]. Two experienced neuropathologists, XL and YJH, each with 10 years of experience in neuropathology, independently reviewed the histological diagnoses for both the development and independent test datasets. Their reviews were aligned with the 2021 WHO classification of tumors of the central nervous system [15]. Any specimens that did not conform to the 2021 WHO classification underwent a secondary review by other neuropathologists (JD with 30 years of experience). The criteria for immunohistochemical and diagnostic assessments in the independent validation dataset were consistent with those used for the development dataset.

### Facial Photo Preprocessing

**Figure 2:**
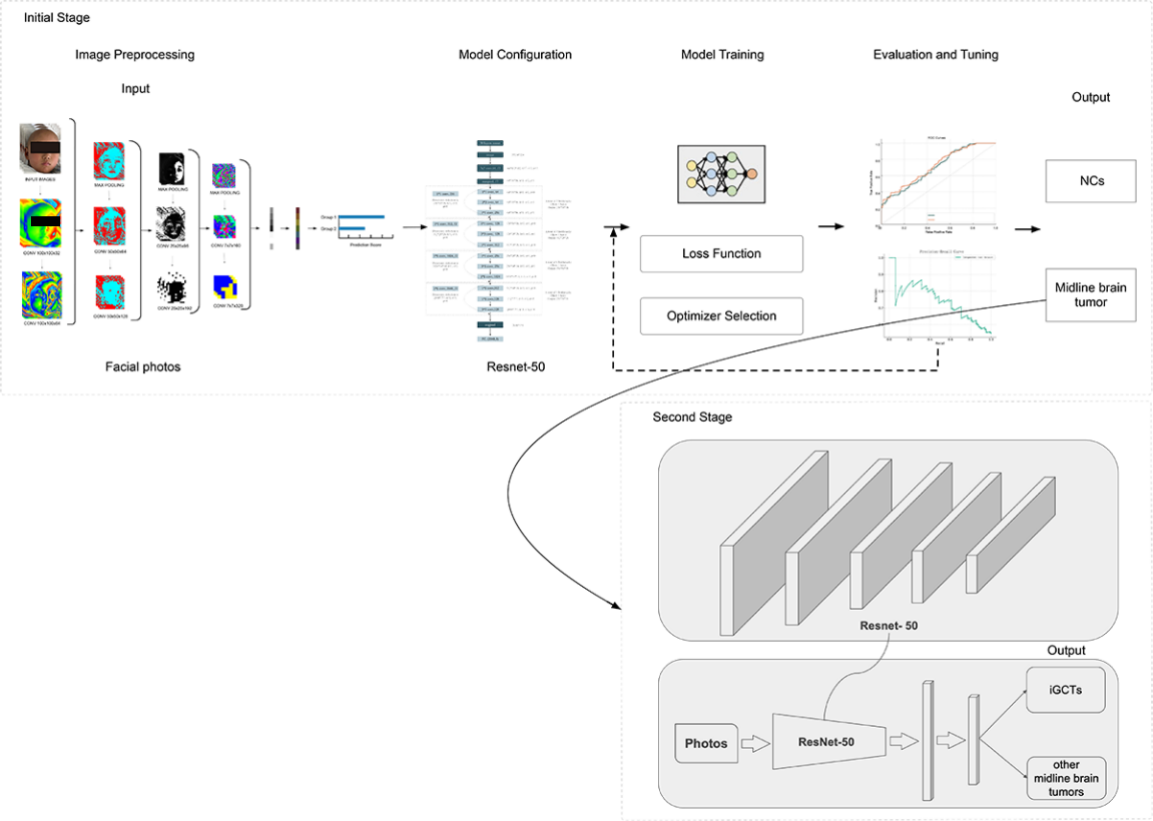
The figure depicts the GVisageNet model workflow for facial photo analysis, including preprocessing, configuration, training with loss function and optimizer selection, and evaluation using ROC and precision-recall curves on an independent external validation data set. iGCTs: intracranial germ cell tumors; NCs: normal controls.

The quality of facial photographs was evaluated by 2 investigators who were blinded to the study design, as outlined in the protocol (Method S2 in Multimedia Appendix 1.). Those that were deemed qualified were processed by cropping to isolate only the facial area, thus removing any excess background and clothing, and were resized to the dimensions of 256×256 pixels. Figure 2 depicts the ResNet-50 model workflow for facial photo analysis, including preprocessing, configuration, training with loss function and optimizer selection, and evaluation using receiver operating characteristic (ROC) and precision-recall curves on an independent external test dataset.

### Facial Recognition Deep Learning Model Development

#### DL Model Architecture

Our facial recognition DL model (GVisageNet) was primarily based on a Convolutional Neural Network (CNN; Figure S1 in Multimedia Appendix 1). We evaluated 3 of the most commonly used candidate models in the field of facial recognition—AlexNet, ResNet-50, and Inception—with ResNet-50 emerging as the most effective through comparative analysis. The development dataset was randomly divided into training and testing datasets in a 75:25 ratio. We used the area under the curve (AUC) to evaluate the classification performance on the testing dataset. To address sample imbalance, resampling techniques were applied to the training dataset, as detailed in Method S3 in Multimedia Appendix 1.

#### Development and Validation of the Facial Recognition DL Model

We selected ResNet [16], specifically ResNet-50 [17], as our model due to its training efficiency. ResNet-50 is composed of 4 blocks, each containing CNN layers of various depths, culminating in a total of 50 CNN layers (Figure S2 in Multimedia Appendix 1). A fully connected layer was added following ResNet-50 for classification purposes. During training, images were resized to 256×256 pixels. Each patient’s facial photos were combined into a 12-channel image stack, ensuring a comprehensive representation of facial features. The supervised model underwent 10 training epochs, using Adam as the optimizer. The initial learning rate was set at 1e^–4^ and adjusted through cosine learning rate decay. The loss function used was cross-entropy. Training was conducted on the training dataset and testing was performed on the testing dataset. Upon completion, the DL model produced probabilities for each image belonging to one of 2 categories, with the highest probability determining the category label. The DL framework was based on PyTorch and operated on an Ubuntu 18.04 system equipped with an NVIDIA V100 Tensor Core GPU. Method S4 in Multimedia Appendix 1 shows the details of the development and validation ResNet-50 based DL model.

#### Evaluating the DL Model

We developed 2 additional models to detect iGCTs, aiming to compare their performance with our primary model. First, we constructed a multivariable logistic regression model using 7 clinical variables, which showed statistical significance in the training dataset as detailed in Table 1. Second, we built a hybrid model that integrates the output of GVisageNet with the results from the logistic regression model, Figure S3 in Multimedia Appendix 1. Both differential diagnostic models underwent training on the training dataset, testing on the testing dataset, and validation on the independent validation dataset.

**Table 1:** The demographic data, symptoms, and endocrine hormone levels of patients across all datasets.

Variable				Development dataset															Independent external validation dataset				
				Training dataset (n=547)							Testing dataset (n=142)							(n=236)					
				Intracranial germ cell tumors (n=358)		Other midline brain tumors (n=189)		*P* values		Intracranial germ cell tumors (n=79)			Other midline brain tumors (n=63)		*P* values		Intracranial germ cell tumors (n=130)				Other midline brain tumors (n=106)		*P* values
**Demographics**																							
		Age (years), mean (SD)	16.95 (8.01)		10.47 (4.72)		.31		15.61 (5.86)			10.61 (4.48)		.42		16.29 (5.49)				14.67 (4.45)		.76	
**Sex**																							
	Female: Male, n:n		167:191		109:80		.52		51:68			33:30		.67		72:58				61:45		.72	
	Duration (months)		15.40		19.60		.39		18.20			17.50		.62		14.60				23.40		.15	
	BMI		18.28		18.59		.88		19.64			16.44		.50		15.82				17.66		.50	
	Primary tumor site (Sellar: Pineal: Fourth ventricle), n:n		186:160:12		89:59: 41		.40		42:31:6			19:29:15		.87		59:61:10				47:51:8		.37	
**Symptoms**																							
	Symptoms of cranial hypertension (headache, vomiting, papilledema), %		76.33		67.42		.41		61.43			52.29		.32		47.66				58.72		.66	
	Vision changes (vision loss, visual field loss), %		43.24		33.21		.56		39.43			48.21		.62		29.56				36.77		.47	
	Growth retardation, %		27.64		10.21		—^a^		—			—		—		—				—		—	
	Limb movement disorder, %		19.88		5.31		<.01^b^		25.66			7.62		.018^b^		17.68				10.32		.22	
**Hypothalamic syndrome, %**																							
	Diabetes insipidus		44.56		21.01		.03^b^		40.98			17.66		<.01^b^		38.21				21.44		.34	
	Abnormal feeding		9.61		7.77		.24		11.43			16.59		.11		20.44				17.43		.52	
	Sleep-wake abnormality		11.21		10.33		.11		20.42			9.61		.03^b^		25.33				11.64		.02^b^	
	Sexual abnormality		8.89		4.23		.04^b^		11.56			6.33		.22		28.42				10.21		.03^b^	
	Affective disturbance		33.21		11.21		.04^b^		29.56			9.43		<.01^b^		19.08				22.44		.54	
	Convulsive seizure		21.99		9.82		.05^b^		36.99			6.55		<.01^b^		18.42				10.33		.08	
**Endocrine hormone levels**																							
	Corticotropic hormone (ACTH), pg/mL		26.63		22.07		.42		22.54			24.67		.78		21.33				19.06		.13	
	Cortisol (COR), μg/dL		7.44		14.32		.07		6.79			12.03		.05^b^		9.65				13.42		.24	
	Thyroid stimulating hormone, mIU/L		0.57		2.33		.02^b^		1.02			3.46		.06		0.99				2.77		<.01^b^	
	Free triiodothyronine, ng/dL		0.77		1.23		.13		0.39			1.45		.08		1.55				2.47		.08	
	Free thyroxine, ng/dL		1.22		1.79		.452		1.44			1.87		.459		0.99				1.68		.09	
	Growth hormone, ng/dL		8.55		21.43		.03^b^		10.22			25.64		.02^b^		9.08				22.37		<.01^b^	
	Insulin-like growth factor-1, ng/mL		189.35		246.88		.48		207.56			255.64		.64		125.42				366.90		.04^b^	
	Insulin-like growth factor binding protein-3, mcg/mL		2.33		5.63		.21		3.42			4.55		.64		1.99				4.43		.07	
	Follicle-stimulating hormone, IU/mL		3.56		7.58		.42		4.43			5.46		.79		3.31				5.73		.57	
	Luteinizing hormone, IU/L		0.43		0.68		.43		0.58			0.77		.64		0.46				0.37		.68	
	Testosterone, ng/dL		78.63		87.43		.88		66.42			69.02		.67		54.37				80.09		.08	
	Estradiol, pg/mL		99.45		121.44		.67		122.44			109.78		.78		133.21				90.76		.29	
	Progesterone, ng/mL		3.77		6.42		.23		5.33			5.89		.82		8.98				6.77		.75	
	Prolactin, μg/L		37.88		33.42		.80		40.66			37.42		.69		29.88				45.53		.35	

^a^Not applicable.

^b^Indicate statistical difference.

The *P* values were determined by the one-way analysis of variance test for age and tumor volume, and the chi-square test for gender, T1WI performance, T2WI performance, enhancement appearances, degree of enhancement, hemorrhage, and necrosis.

#### The Working Mechanism of Interpretation Model Potential

To explore the hypothesis that endocrine changes contribute to alterations in facial characteristics, we conducted a correlation analysis between prediction scores and endocrine indicators. This analysis focused on the independent validation dataset, aimed to uncover the underlying mechanisms. In addition, we segmented the independent validation dataset into 3 subgroups based on the extent of HPT axis damage: complete damage, incomplete damage, and no damage, Method S5 in Multimedia Appendix 1 defines the “complete damage,” “incomplete damage,” and “no-damage” of the HPT axis damage. The performance of GVisageNet was then compared across these subgroups to determine if the degree of endocrine damage influenced the model’s effectiveness.

### Statistical Analysis

For our statistical analysis, we used SPSS (version 25.0) software (IBM Corp). Continuous variables were presented as mean (SD), and discrete variables as percentages. We assessed the normality of continuous variables using the Shapiro-Wilk test. Depending on the results, either the independent samples *t* test, Friedman test, or the Mann-Whitney *U* test was used for comparison. Categorical variables, such as gender and endocrine abnormalities in patients with NCs and midline brain tumors (including iGCTs and other midline brain tumors), were analyzed using the chi-square test. We calculated the 95% exact CIs for both proportions and mean differences.

To evaluate the performance of the DL model, we computed its diagnostic accuracy, sensitivity, specificity, and the area under the ROC curve, using pathological diagnosis as the benchmark. The model’s performance was evaluated at 2 specific points on the receiver operating characteristic ROC curve, chosen for their maximum sensitivity and specificity. We used the Delong test [18] to compare the AUCs of the different models. Multinomial logistic regression was used to examine the correlation between the prediction scores and the endocrine indicators. All comparisons were conducted as 2-sided tests, with a *P* value of less than .05 considered statistically significant.

### Ethical Considerations

Ethical approval was granted by the institutional review board of Beijing Tiantan Hospital, Capital Medical University (KY2021-142-02). Informed consent was secured from all patients or their guardians. Privacy and Confidentiality: In this study, all participant data, including images, are processed with strict measures to ensure privacy and confidentiality. Each participant’s data, including facial photographs, is anonymized and deidentified before analysis to prevent any direct or indirect identification. For cases where anonymization is not feasible, such as if the integrity of facial features is required for model training or analysis, robust data protection protocols are implemented. These include secure storage solutions, restricted access, and ethical review board oversight, ensuring compliance with data privacy standards.

Compensation details are that the participants involved in the study were compensated fairly for their contributions. All individuals provided informed consent, detailing the nature and amount of compensation received, proportional to the time and effort required for their participation, in accordance with ethical guidelines.

## Results

### Patient Characteristics

**Figure 3:**
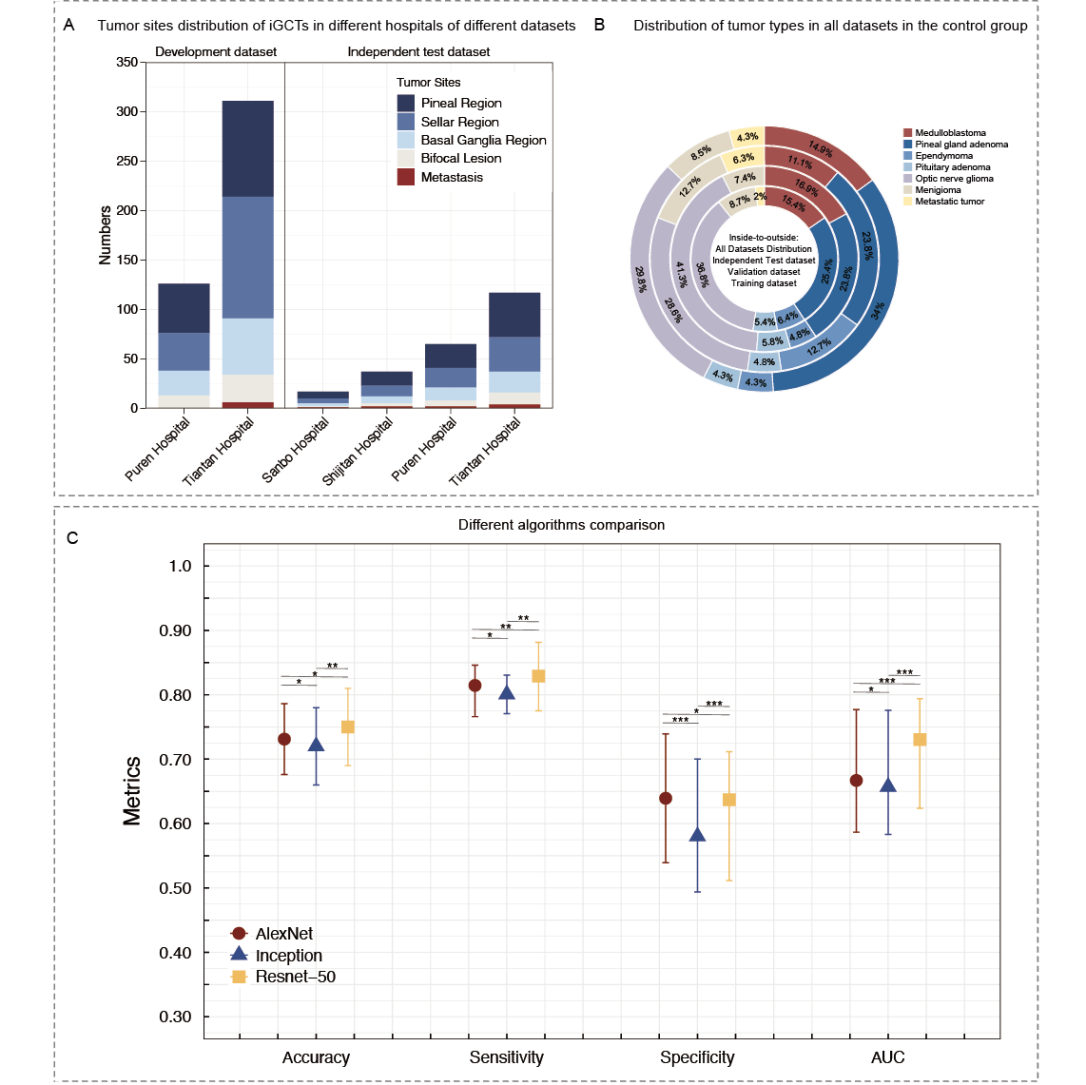
(A) Tumor site distribution of iGCTs in different hospitals of different datasets. (B) Distribution of tumor types in all datasets in the other midline brain tumors group. (C) Comparison of performance of different algorithms (accuracy, sensitivity, specificity, and area under the curve). AUC: area under the curve; iGCTs: intracranial germ cell tumors.

Initially, we obtained frontal facial photographs of 989 subjects (including 300 NCs, 437 patients with iGCTs, and 252 patients with other midline brain tumors). These photos were collected for the training and testing of the DL model with facial recognition, GVisageNet, from January 2010 to December 2020 at 2 medical institutes. In addition, an independent validation dataset, comprising 200 NCs, 130 patients with iGCTs, and 106 patients with other midline brain tumors, was collected from four medical institutes between January 2021 and May 2023 for validation of the DL model. The demographic data, symptoms, and endocrine hormone levels of all patients across all datasets (training, testing, and independent validation) are summarized in Table 1. The training, testing and independent validation datasets included 500 NCs (250 males and 250 females, with mean age of 13.00, SD 5.15 years), 567 patients with iGCTs (256 males and 290 females, with a mean age of 12.81, SD 5.79 years) and 358 patients with other midline brain tumors 155 males and 203 females, with a mean age of 13.61, SD 4.93 years), respectively. We filtered 7 clinical variables (limb movement disorder, diabetes insipidus, sexual abnormality, affective disturbance, convulsive seizure, TSH, and GH) that were statistically different between the iGCTs group and other midline brain tumors group in the training/testing dataset. Figures 3A and 3B illustrate the distribution of iGCT tumor sites across various hospitals in different datasets, as well as the distribution of tumor types within all datasets among other midline brain tumor groups.

### Evaluation of Three Candidate Algorithmic Models

In this investigation, we conducted a comparative analysis of 3 distinct candidate algorithms by training each on the training dataset and then evaluating their respective performances on the testing dataset. The algorithms tested were AlexNet, ResNet-50, and Inception. In the testing dataset, GVisageNet and the other models (AlexNet and Inception) yielded similar results in the first stage (distinguishing patients with midline brain tumors and NCs) with AUCs of 0.972 (95% CI 0.951-0.992), 0.963 (95% CI 0.943-0.984), and 0.983 (95% CI 0.937-1.000), respectively, and *P*>.05. Therefore, we proceeded to compare the results from the second stage. In the second stage, based on the AUC values with 95% CIs, the following results were observed: AlexNet achieved an AUC of 0.667 (95% CI 0.582-0.777), ResNet-50 reached an AUC of 0.705 (95% CI 0.590-0.760), and Inception yielded an AUC of 0.657 (95% CI: 0.583-0.776; Figure 3C and Table 2). Given these outcomes, ResNet-50 emerged as the most proficient model in terms of classification performance and was subsequently chosen as the final model for our classification tasks. Table S1 in Multimedia Appendix 1 and Figure S2 in Multimedia Appendix 1 presented additional details.

**Table 2:** The AUC, accuracy, sensitivity, and specificity of the different model of testing and independent external validation datasets.

Model	Dataset	AUC^a^ (95% CI)	Accuracy (95% CI)	Sensitivity (95% CI)	Specificity (95% CI)
GVisageNet	Testing	0.705 (0.590-0.760)	71.71% (57.42%-77.11%)	75.24% (70.89%-77.57%)	73.40% (69.04%-76.80%)
GVisageNet	Independent validation	0.739 (0.728-0.773)	73.10% (69.85%-77.34%)	68.33% (53.65%-75.14%)	63.24% (56.59%-81.20%)
Logistic regression	Testing	0.612 (0.493-0.709)	59.41% (50.85%-64.28%)	65.18% (56.78%-73.54%)	55.93% (48.48%-65.74%)
Logistic regression	Independent validation	0.632 (0.571-0.679)	68.97% (62.68%-75.44%)	57.88% (49.45%-68.74%)	58.59% (49.24%-70.04%)
Hybrid model	Testing	0.711 (0.676-0.798)	70.77% (65.48%-76.65%)	72.94% (66.99%-76.84%)	67.78% (66.31%-72.53%)
Hybrid model	Independent validation	0.789 (0.728-0.793)	71.56% (68.90%-73.84%)	71.77% (56.48%-76.88%)	58.45% (50.73%-66.05%)

^a^AUC: area under the curve.

### Performance of GVisageNet

In the testing dataset, GVisageNet achieved an AUC of 0.942 (95% CI 0.921-0.972), an accuracy of 94.33% (95% CI: 91.00%-95.96%), a sensitivity of 93.51% (95% CI: 90.00%-96.50%), and a specificity of 93.39% (95% CI 89.48%-97.00%) in distinguishing NCs from midline brain tumors. In second stage, GVisageNet achieved an AUC of 0.705 (95% CI 0.590-0.760), an accuracy of 71.71% (95% CI 57.42%-77.11%), a sensitivity of 75.24% (95% CI 70.89%-77.57%), and a specificity of 73.40% (95% CI 69.04%-76.80%) in distinguishing iGCTs from other midline brain tumors.

**Figure 4:**
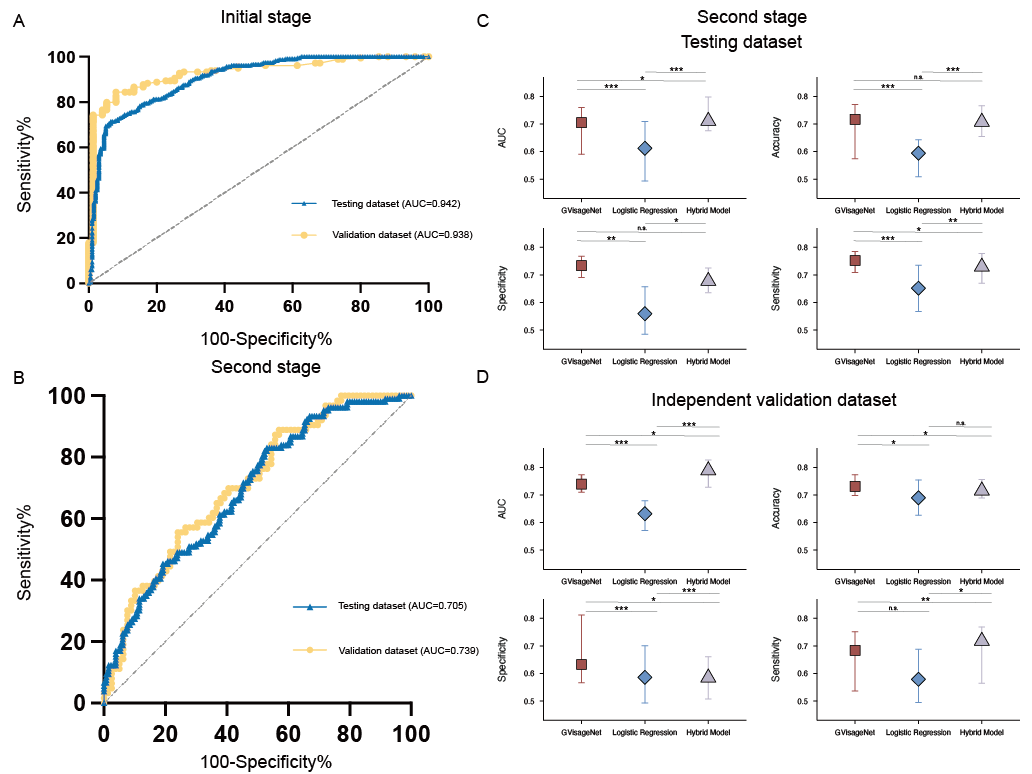
(A) The receiver operator characteristic curve of GVisageNet in the initial stage of development in the testing and independent external validation datasets. (B) The receiver operator characteristic curve of GVisageNet in the second stage of development in the testing and independent external validation datasets. (C) The area under the curve, accuracy, sensitivity, and specificity of the GVisageNet in the testing dataset with 95 % CI. (D) The AUC, accuracy, sensitivity, and specificity of the GVisageNet in the independent external validation dataset with 95% CI. AUC: area under the curve.

In the independent external validation dataset, GVisageNet demonstrated effectiveness in distinguishing NCs and midline brain tumors. It achieved an AUC of 0.938 (95% CI 0.913-0.967), an accuracy of 83.77% (95% CI: 71.45%-92.33%), a sensitivity of 91.67% (95% CI 87.42%-100.00%), and a specificity of 77.55% (95% CI 66.43%-80.46%; Figure 4A). In distinguishing iGCTs from other midline brain tumors, as shown in Figure 4B, it achieved an AUC of 0.739 (95% CI 0.728-0.773), an accuracy of 73.10% (95% CI 69.85%-77.34%), a sensitivity of 68.33% (95% CI 53.65%-75.14%), and a specificity of 63.24% (95% CI 56.59%-81.20%). The AUC, accuracy, sensitivity, and specificity of GVisageNet in the testing and validation datasets of the second stage are depicted in Figures 4C and 4D.

### Clinical Variables and Hybrid Model Performance in Differentiating iGCTs From Other Midline Brain Tumors

To enhance GvisageNet’s ability to distinguish iGCTs from other midline brain tumors, we compared the performance of a logistic regression model using clinical variables with a hybrid model. In the testing dataset, the logistic regression model, which used clinical variables, achieved an AUC of 0.612 (95% CI 0.493-0.709), an accuracy of 59.41% (95% CI 50.85%-64.28%), a sensitivity of 65.18% (95% CI 56.78%-73.54%), and a specificity of 55.93% (95% CI 48.48%-65.74%). In the independent external validation dataset, this model attained an AUC of 0.632 (95% CI 0.571-0.679), an accuracy of 68.97% (95% CI 62.68%-75.44%), a sensitivity of 57.88% (95% CI 49.45%-68.74%), and a specificity of 58.59% (95% CI: 49.24%-70.04%), as depicted in Figures 4C and 4D, and Table S2 in Multimedia Appendix 1.

The hybrid model in the testing dataset achieved an AUC of 0.711 (95% CI 0.676-0.798), an accuracy of 70.77% (95% CI 65.48%-76.65%), a sensitivity of 72.94% (95% CI 66.99%-76.84%), and a specificity of 67.78% (95% CI 66.31%-72.53%). In the independent external validation dataset, the hybrid prediction model reached an AUC of 0.789 (95% CI 0.728-0.793), an accuracy of 71.56% (95% CI 68.90%-73.84%), a sensitivity of 71.77% (95% CI 56.48%-76.88%), and a specificity of 58.45% (95% CI 50.73%-66.05%), as shown in Figures 4C and 4D.

In a comparative analysis conducted on the independent external validation dataset, GVisageNet was found to significantly outperform the logistic regression model, demonstrating higher accuracy (AUC 0.739 vs 0.632; *P*<.001). However, its performance was slightly inferior to that of the hybrid model (AUC 0.739 vs 0.789; *P*=.04).

### Correlation Analysis Between Prediction Output and Endocrine Indicators

**Figure 5:**
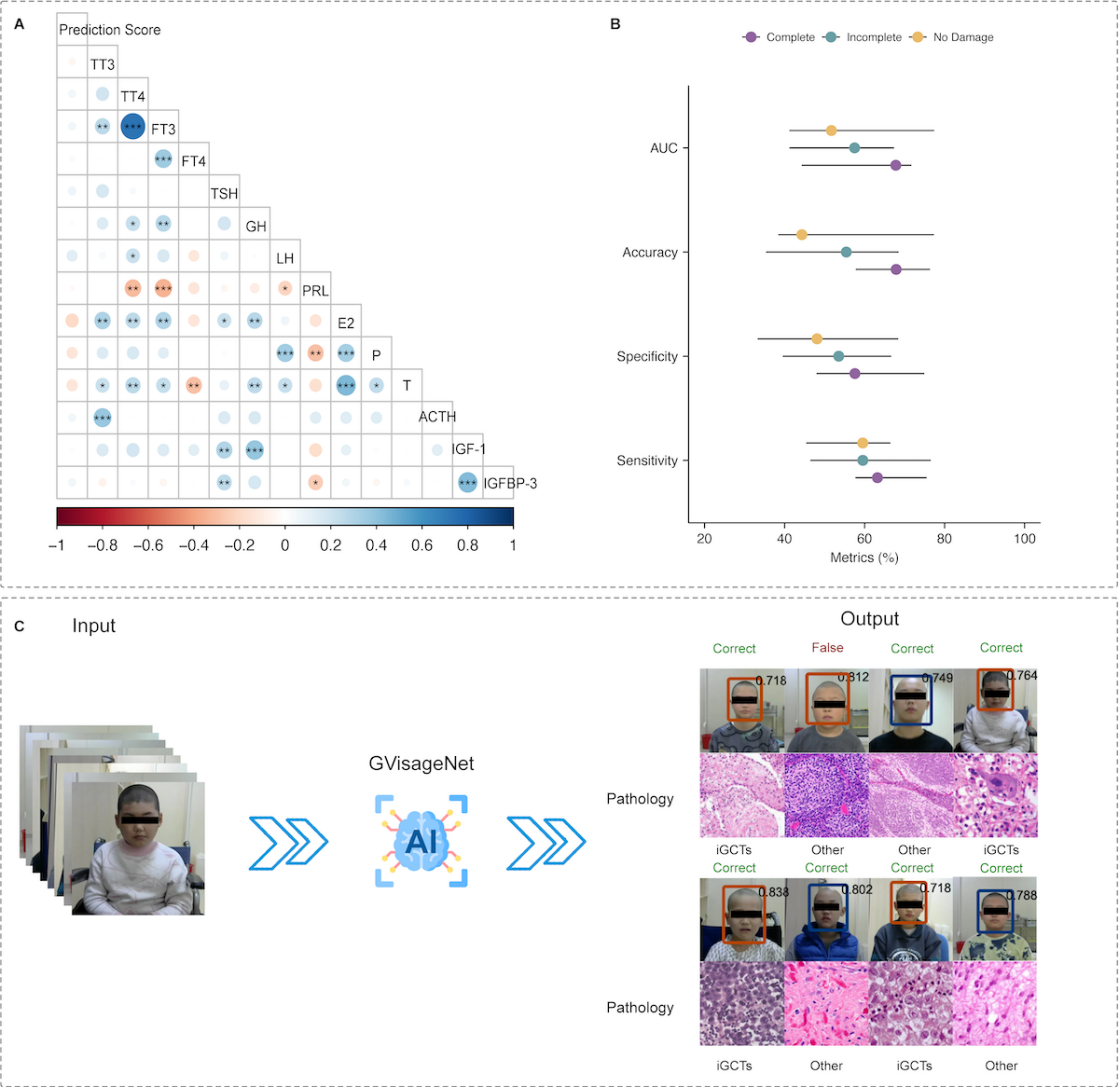
(A) Correlation analysis between the output results of GVisageNet and endocrine indicators in the independent external validation dataset. (B) The area under the curve, accuracy, sensitivity, and specificity of the GVisageNet for the stratified analysis of the HPT axis damage degree in the independent external validation dataset. (C) The visual integration of GVisageNet into the facial photo collection system (independent validation dataset). ACTH: adrenocorticotrophic hormone; E2: estradiol; FT3: free triiodothyronine; FT4: free thyroxine; GH: growth hormone; iGCTs: intracranial germ cell tumors; IGF-1: insulin-like growth factor 1; IGFBP-3: insulin-like growth factor binding protein 3; LH: luteinizing hormone; P: progesterone; PRL: prolactin; T: testosterone; TSH: thyroid-stimulating hormone; TT3: total tri-iodothyronine; TT4: total thyroxine.

Correlation analysis revealed a significant positive correlation between the prediction scores and the levels of TSH and IGFBP-3 (*P*<.05). Conversely, there was a significant negative correlation between the prediction scores and the levels of total thyroxine (TT4), FT3, FT4, GH, prolactin, and estradiol (*P*<.05). Detailed pairwise concordances of endocrine indicators are presented in Figure 5A.

Moreover, the independent validation dataset was divided into 3 groups based on the degree of HPT axis damage: complete damage (n=37), incomplete damage (n=54), and no damage (n=39). In the task of differentiating iGCTs from other midline brain tumors, GVisageNet achieved varying levels of performance across these groups. In the complete damage group, GVisageNet achieved an AUC of 0.678 (95% CI 0.443-0.717), an accuracy of 67.89% (95% CI 57.79%-76.33%), a sensitivity of 63.21% (95% CI 57.67%-75.54%), and a specificity of 57.58% (95% CI 47.99%-74.91%). For the incomplete damage group, the model reached an AUC of 0.575 (95% CI 0.412-0.674), an accuracy of 55.43% (95% CI 35.37%-68.54%), a sensitivity of 59.56% (95% CI 46.43%-76.54%), and a specificity of 53.54% (95% CI 39.54%-66.64%). In the no-damage group, GVisageNet achieved an AUC of 0.517 (95% CI 0.412-0.774), an accuracy of 44.32% (95% CI 38.43%-77.33%), a sensitivity of 59.54% (95% CI 45.43%-66.47%), and a specificity of 48.11% (95% CI 33.23%-68.42%; Table 3). The AUC, accuracy, sensitivity, and specificity for each group are detailed in Figure 5B. An analysis of variance (Friedman test) revealed a significant difference in AUC among the 3 groups (*P*=.03). The visual integration of GVisageNet into the facial photo collection system is shown in Figure 5C.

**Table 3:** The AUC, accuracy, sensitivity, and specificity of the GVisageNet in the independent external validation dataset for the stratified analysis of the HPT axis damage degree.

Dataset	AUC^a^ (95% CI)	Accuracy (95% CI)	Sensitivity (95% CI)	Specificity (95% CI)
Complete damage	68.00% (44.30%-71.70%)	67.89% (57.79%-76.33%)	63.21% (57.67%-75.54%)	57.58% (47.99%-74.91%)
Incomplete damage	57.00% (41.20%-67.40%)	55.43% (35.37%-68.54%)	59.56% (46.43%-76.54%)	53.54% (39.54%-66.64%)
No-damage	52.00% (41.20%-77.40%)	44.32% (38.43%-77.33%)	59.54% (45.43%-66.47%)	48.11% (33.23%-68.42%)

^a^AUC: area under the curve.

## Discussion

### Principal Findings

This study successfully developed and validated an automated binary DL model, GVisageNet, to differentiate midline brain tumors from NCs and further distinguish iGCTs from other midline brain tumors using facial images. We also constructed a logistic regression model and a hybrid model using clinical data to explore whether clinical variables could enhance the diagnostic performance of the model. The results showed that in differentiating iGCTs from other midline brain tumors, the hybrid model slightly outperformed GVisageNet (0.739 vs 0.789; *P*=.04). Finally, we attempted to explain the mechanism of GVisageNet by analyzing endocrine changes in patients and found that the model’s predicted probability values were strongly correlated with seven endocrine indicators (*P*<.05). In addition, GVisageNet performed best in the group with complete endocrine axis damage, suggesting that endocrine axis impairment may be a reason for the facial texture changes observed in patients. GVisageNet can be integrated into outpatient facial photo collection systems, enabling the routine collection of facial images during a patient's initial visit. This allows for real-time preliminary screening diagnoses, guiding clinicians to conduct targeted examinations, thereby saving medical resources and reducing patient costs.

The enhanced ResNet-50 model demonstrated significant advancements in this field, outperforming both its original version and other conventional CNN models in terms of training and testing accuracy. This improvement was achieved through structural modifications and an optimized training process, including an adaptive learning rate that effectively adjusts weights within the network layers. Moreover, the model effectively addressed the common issue of overfitting in DL, thereby enhancing its reliability and consistency in screening applications. A crucial aspect of this improvement was the model’s reduced loss values and fluctuations, contributing to its increased stability. Importantly, visualization tools were used for feature extraction from specific layers of ResNet-50 [19], enabling precise identification of potential tumor indicators from facial photographs [20], which is essential for early screening and intervention. In addition, the introduction of a standard operating procedure for preprocessing facial photos significantly improved the quality of input data, aiding in more accurate tumor screening.

In addition to the analyses conducted, it is essential to consider the potential issue of collinearity among features. Collinearity, or high intercorrelation among predictor variables, can adversely affect the performance and interpretability of regression models and other machine learning techniques. Detecting and addressing collinearity is crucial to ensure the robustness of our models. Methods such as correlation matrices, variance inflation factors, and condition indices can be used to detect collinearity [21]. Mitigation strategies include removing or combining highly correlated features and using regularization techniques like Ridge regression or Lasso [22]. Future work should include a thorough investigation of collinearity and its impact on model performance. By addressing collinearity, we can enhance the stability and interpretability of our models, leading to more reliable diagnostic predictions. Therefore, it is recommended that future studies incorporate collinearity diagnostics and report any steps taken to mitigate its effects. This approach will provide a more comprehensive understanding of the model development process and ensure the robustness of the predictive models.

Furthermore, DL models, especially those designed for facial recognition, are adept at extracting and analyzing complex image patterns that may reflect physiological changes due to endocrine imbalances. Several mechanisms underlie the potential correlations between DL model outputs and endocrine indicators. First, models like CNNs can identify subtle changes in facial features associated with hormonal imbalances, such as those caused by conditions like acromegaly or Cushing’s syndrome, which alter fat distribution, skin texture, or facial bone structure. Second, these models use extensive training datasets to recognize patterns associated with specific conditions, such as the protruding eyes or facial puffiness seen in hyperthyroidism, and can correlate these visual cues with hormone levels (eg, TSH, triiodothyronine, and thyroxine) to predict endocrine states from the facial analysis. In addition, hormones significantly affect physical characteristics, where elevated cortisol levels may cause a “moon face” appearance or a buffalo hump, and excess growth hormone can change the jawline and brow prominence, all detectable through DL analysis. Finally, integrating DL outputs with clinical data, such as hormone levels, enhances predictive accuracy. A hybrid model that merges image-based phenotypic features with biochemical markers provides a comprehensive understanding of a patient’s endocrine state, enabling nuanced predictions and personalized health assessments. This integrated approach leverages facial recognition technology to noninvasively assess endocrine conditions, thereby enhancing diagnostic precision and patient care.

In addition, exploring the potential broader applications of our DL model in diagnosing other diseases with distinct phenotypic manifestations is imperative. For instance, conditions such as Parkinson disease, characterized by specific facial expressions and muscle rigidity, or genetic disorders like Down syndrome, which presents with unique facial features, could benefit from analogous DL-based diagnostic approaches [23,24]. By training models to recognize these specific phenotypic patterns, we can expand the use of our DL model to provide early and accurate diagnoses across a variety of medical conditions. This broader application not only enhances the model’s relevance in the medical field but also contributes to more comprehensive patient care through the early detection of diseases that manifest through observable physical changes.

Significant correlations were observed between DL model outputs and endocrine markers, including TSH, IGFBP-3, TT4, FT3, FT4, GH, prolactin, and estradiol, suggesting that endocrine variations may play a crucial role in the development of facial features in patents with iGCT. Notably, approximately 40% of iGCTs secrete β-HCG [1,5,25], which also induces endocrine changes at sufficiently high levels. The cross-immunoreactivity of β-HCG with LH triggers peripheral precocious puberty or gonadotropin-releasing hormone–independent precocious puberty, distinct from central precocious puberty and nonprogressive pubertal variants. In males, elevated β-HCG levels activate LH receptors on Leydig cells, enhancing testosterone production [26]. Conversely, in females, β-HCG does not induce precocious puberty, as activation of both FSH and LH receptors is requisite. Consequently, precocious puberty in iGCTs is predominantly observed in male toddlers and school-aged boys, characterized by facial features such as acne, beard growth, increased oil secretion, and skeletal maturity. Excess GH production leads to acromegaly [27], resulting in enlarged facial bones, while hypothyroidism causes facial puffiness [28-32], especially around the eyes [33-35]. When DL model results were integrated with clinical indicators, the detection efficacy was significantly enhanced. It is imperative to clarify that β-HCG was excluded from our study since only 40% of patients exhibit β-HCG secretion, and the DL model serves as a prediagnostic screening tool, hinting at further examination. Premature inclusion of tumor markers could compromise model accuracy. This observation was further underscored in the stratified analysis. Our findings indicate that the predictive efficacy of GVisageNet was least effective in cases without HPT axis impairment, and most effective in instances of complete HPT axis disruption. This underscores a direct correlation between the model's predictive capacity and endocrine function. Notably, when the HPT axis was fully compromised, the resultant endocrine-driven alterations in facial characteristics were most pronounced.

This study had several limitations that should be considered when interpreting the findings. First, the relatively small sample size of each iGCT subtype (germ cell tumors [germinomas] and nongerminomatous germ cell tumors) limited the ability to conduct a more detailed analysis and might have impacted the statistical power of the study. Future research should aim to include larger cohorts to enhance the robustness and generalizability of the findings. Second, the study only collected baseline facial photos of the patients. The lack of longitudinal follow-up data limits the ability to observe changes in facial features and endocrine parameters over time. Longitudinal studies are needed to evaluate the progression and potential predictive value of facial recognition technology in monitoring patients with iGCTs. Third, the study was conducted across multiple centers, which introduced variability in data collection and image quality. Although efforts were made to standardize procedures, some degree of inconsistency is inevitable. Future studies should implement stricter protocols and quality control measures to minimize such variability. Finally, while the study focused on differentiating iGCTs from other midline brain tumors, it did not explore the potential of this technology in distinguishing iGCTs from other types of intracranial tumors or nontumorous conditions. Expanding the scope of future research could provide a more comprehensive understanding of the clinical applications of deep learning-based facial recognition in neuro-oncology.

In addition, “no identifiable personal information” (such as unique facial characteristics) is presented in any figures or supplementary materials of the manuscript. In cases where participant consent is necessary for image inclusion, signed consent forms have been obtained and can be uploaded in the “Other files” field upon resubmission. This ensures transparency and adheres to the highest standards of ethical research practices.

### Conclusion

In summary, this study demonstrates the potential application of facial recognition technology in the diagnosis of iGCTs. Facial recognition could not only aid in early detection and screening of iGCTs but also might become a valuable tool in advancing future clinical decision-making.

## Appendix

Multimedia Appendix 1 Additional methods, figures, and tables for the protocol.

## Abbreviations

AUC: area under the curve
CNN: convolutional neural network
DL: deep learning
FSH: follicle-stimulating hormone
FT3: free triiodothyronine
FT4: free thyroxine
GH: growth hormone
HCG: human chorionic gonadotropin
HPT: hypothalamic-pituitary-target
iGCT: intracranial germ cell tumor
IGFBP-3: insulin-like growth factor binding protein 3
LH: luteinizing hormone
NC: normal control
PD: panic disorder
ROC: receiver operating characteristic
TSH: thyroid stimulating hormone
TT4: total thyroxine
WHO: World Health Organization
