# Supplementary material for: Identification of Intracranial Germ Cell Tumors Based on Facial Photos: Exploratory Study on the Use of Deep Learning for Software Development
Source: J Med Internet Res. 2025 Jan 30;27:e58760. doi: 10.2196/58760 (PMC11826948; doi:10.2196/58760)
Supplement: Multimedia Appendix 1 [file jmir_v27i1e58760_app1.docx]

**SUPPLEMENTAL MATERIALS**

**Identification of Intracranial Germ Cell Tumors Based on Facial Photo: Software Development Using Deep Learning Technology**

**Contents**

**Supplementary Method1.** Protocol for Facial Photo Collection.

**Supplementary Method 2.** Photo Quality Check and Preprocessing.

**Supplementary Method 3.** Algorithm Construction

**Supplementary Method 4.** The details of development and testing ResNet-50 based DL model.

**Supplementary Method 5.** Defining hypothalamic-pituitary-target organ axis (HPT axis) damage in terms of "complete damage," "incomplete damage," and "no damage".

**Supplementary Figure 1.** Illustration of CNN-based classifier.

**Supplementary Figure 2.** Deep Neural Network Architecture.

**Supplementary Figure 3.** Illustration of CNN-based hybrid model.

**Supplementary Table 1.** The diagnosis of other midline brain tumors.

**Supplementary Table 2.** The 95% confidence interval of all diagnostic measurements.

**Method S1.** Protocol for Facial Photo Collection.

Facial photos were collected according to the following standardized protocol:

1. Time: Within 2 week of the diagnosis of intracranial germ cell tumors (iGCTs).

(2) Camera Setting: Digital cameras with more than 10 million pixels, P mode, camera sensitivity 1600, burst mode.

(3) Photo Background: Pure white, direct light.

(4) Patient Preparation: Eyes open, no facial expression, no accessories, and no hair covering any facial features.

(5) Photograph: A trained investigator blinded to the study design should take frontal photographs of the patient’s face.

(6) Preliminary Quality Check: The photo should cover the area between the top of the head, ears, and the area equivalent to a man’s prominentia laryngea. The whole head should be clearly seen, especially the ear contour and forehead.

**Method S2.** Photo Quality Check and Preprocessing.

(1) Photo Quality Check

Facial photos were checked by two investigators blinded to the study design to ensure uniform photo quality and reduce noise. The photos were defined “**qualified**” if they met all of the items below. In contrast, the photos which failed to meet any of these items were defined as “**unqualified**”. Unqualified photos were excluded from final analysis.

1. Angles: Frontal photos.

2. Moderate light: The light should not be too dark or too light to make sure no missing information in facial features.

(2) Computer photo preprocessing

Qualified facial photos underwent further preprocessing using software to ensure uniformity of photo quality. Detailed photo preprocessing guidelines are described below.

1. All photos were cropped using computer software to remove excess background and clothing, retaining only the face in the photos.

2. Photos were resized to a uniform 256×256 pixels.

**Method S3.** Algorithm Construction.

We aimed to develop an intelligent diagnostic approach that could autonomously extract relevant information from facial images for predicting the presence of iGCTs. We used DL because it has achieved remarkable success and shown strong ability in various computer vision tasks. The iGCTs prediction was considered a binary classification problem in our study, and we built a deep classification model for it. The model learned to extract disease-related features from the raw input and made suitable classification decisions based on these features. The model was trained in an end-to-end manner, which has been verified as an effective approach in multiple tasks involving computer vision.

To construct our algorithm, we employed deep CNNs^1^. CNN-based classifiers provide a state-of-the-art approach to vision-based classification tasks. The classifier in our study consisted of a sequence of convolutional and fully-connected layers. All layers were stacked for cascaded data processing. The classification process occurred in two stages: I. Feature Extraction, and II. Classification.

**Method S4.** The details of development and testing ResNet-50 based DL model.

ResNet^2^ utilizes two types of mappings: identity and residual. Identity mapping, or the "straight path," represents the original input x in the equation y = F(x) + x, while residual mapping, or the "difference," refers to F(x). ResNet-50 begins with a convolution of the input, followed by four residual blocks, culminating in a fully connected (FC) layer for classification. Illustrated in ***Figure. S3***, ResNet-50^3^ incorporates 50 Conv2D operations. The FC layer, typically at the network's end, consolidates features from preceding layers. It can be viewed as a phase of feature weighting, learning a potential nonlinear function. This process starts by transforming the image into a format suitable for a multilevel perceptron, flattening it into column vectors, and then processing it through a feedforward neural network. Each training iteration applies this flattened data, enabling the model to discern primary and subtle features in the image and categorize them using techniques like Softmax.

**Training Process**

During training, the parameters of the model were adjusted to minimize the binary cross-entropy loss, $\mathcal{L}$ (Eq. 1).

$$\mathcal{L=}\sum_{N} \sum_{C} y_{n,c}\cdot log(\tilde{y}_{n,c}) (1)$$

where N was the number of samples, $C$ was the number of classes ($here, C=2$), and $\tilde{y}_{s,c}$ and $y_{s,c}$ represented the predicted probability and the ground-truth probability, respectively, on the class c for the n-th sample. The parameters were updated from the last layer back to the first layer (Eq. 2) with the backpropagation algorithm^4^. Specifically, the $i$-th layer’s parameter, $\theta_{i}$, was adjusted at each step following the minus gradient direction (Eq. 3) to minimize the loss.

$$\mathcal{L\to H\to O\to}\mathcal{F}_{N}\to\ldots\to\mathcal{F}_{i}\to\ldots\to\mathcal{F}_{1} (2)$$

$$\Delta\theta_{i}=-\lambda\frac{\partial\mathcal{L}}{\partial\theta_{i}}=-\lambda(\frac{\partial\mathcal{L}}{\partial\mathcal{H}}\frac{\partial\mathcal{H}}{\partial\mathcal{O}}\frac{\partial\mathcal{O}}{\partial\mathcal{F}_{N}}\frac{\partial\mathcal{F}_{N}}{\partial\mathcal{F}_{N-1}}\cdot\cdot\cdot\frac{\partial\mathcal{F}_{i}}{\partial\theta_{i}}) (3)$$

where $\mathcal{L}$ was the loss, $\mathcal{H}\mathrm{and}\mathcal{O}$ were the softmax and activation functions of the output layer, and $\mathcal{F}_{i}$ represented the function of the $i$-th layer.

In experiments, we used SGD (Stochastic Gradient Descent) with moment 0.9 and weight decay 0.0001. The number of training epochs was 100. The initial learning rate was 0.01, with this rate dropping by 10 after the 50th and 90th epochs. The batch size was 32.

**“Facial Algorithm + Logistic Regression Model” Training**

We built a hybrid model that simultaneously leveraged facial photos and tumor marker variables to predict iGCTs status. The model was constructed by concatenating the tumor marker variables to the fully connected layers as input.

**Method S5.** Defining hypothalamic-pituitary-target organ axis (HPT axis) damage in terms of "complete damage," "incomplete damage," and "no damage".

**No-damage**

Clinical Symptoms: Absence of clinical symptoms indicative of HPT axis dysfunction (e.g., normal growth patterns, normal pubertal development).

Hormonal Levels: Normal levels of hormones associated with the HPT axis, including but not limited to thyroid-stimulating hormone (TSH), adrenocorticotropic hormone (ACTH), follicle-stimulating hormone (FSH), luteinizing hormone (LH), and their respective target organ hormones (e.g., thyroid hormones, cortisol, sex hormones).

Functional Assessments: Normal responses in dynamic testing of the HPT axis (e.g., stimulation tests, suppression tests) indicating intact regulatory feedback mechanisms^4^.

**Incomplete Damage**

Clinical Symptoms: Presence of some, but not all, clinical symptoms indicative of HPT axis dysfunction, which may include mild to moderate growth abnormalities, delayed or precocious puberty, or other subclinical signs.

Hormonal Levels: Hormonal levels that indicate partial dysfunction of the HPT axis, such as slightly elevated or reduced hormone levels that do not conform to the pattern expected for complete axis damage. This may involve abnormal levels of one or more pituitary or target organ hormones, suggesting partial disruption of axis function.

Functional Assessments: Abnormal responses in dynamic testing that suggest a diminished but not absent capacity for hormone regulation and feedback within the HPT axis. This may be indicated by blunted but detectable responses to stimulation or suppression tests.

**Complete Damage**

Clinical Symptoms: Presence of significant clinical symptoms indicative of HPT axis dysfunction, including severe growth abnormalities, significant pubertal delay or failure, severe fatigue, and other symptoms of hormonal deficiency or excess.

Hormonal Levels: Hormonal levels clearly indicative of a failure in the HPT axis, with significant deviations from normal levels. This includes markedly low or high levels of pituitary hormones and their corresponding target organ hormones, suggesting a complete disruption of axis communication and regulation.

Functional Assessments: Lack of appropriate hormonal response in dynamic testing, indicating a failure in the regulatory feedback mechanisms of the HPT axis. This is characterized by absent or near-absent responses to stimulation or suppression tests, confirming the loss of functional axis activity.

**Figure S1.** Illustration of CNN-based classifier.

**
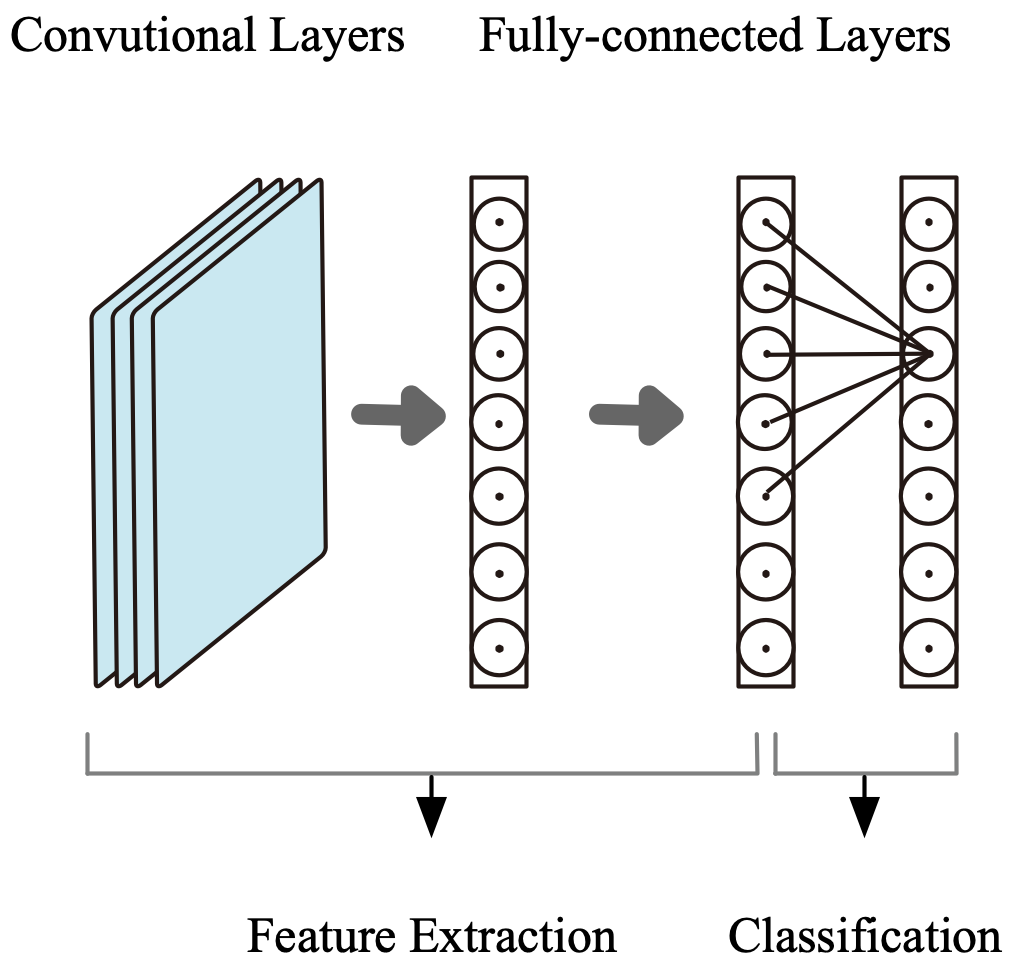
**

**Figure S2.** Deep Neural Network Architecture.

**
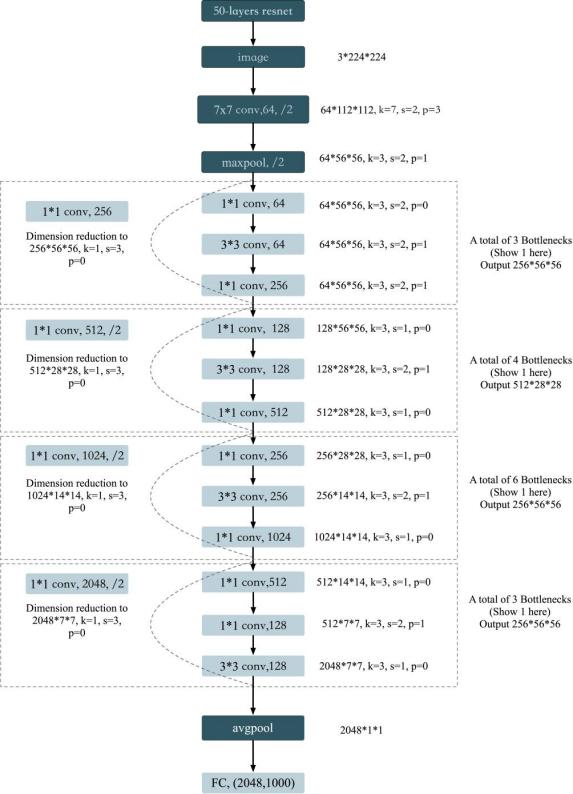
**

**Figure S3.** Illustration of CNN-based hybrid model.

**
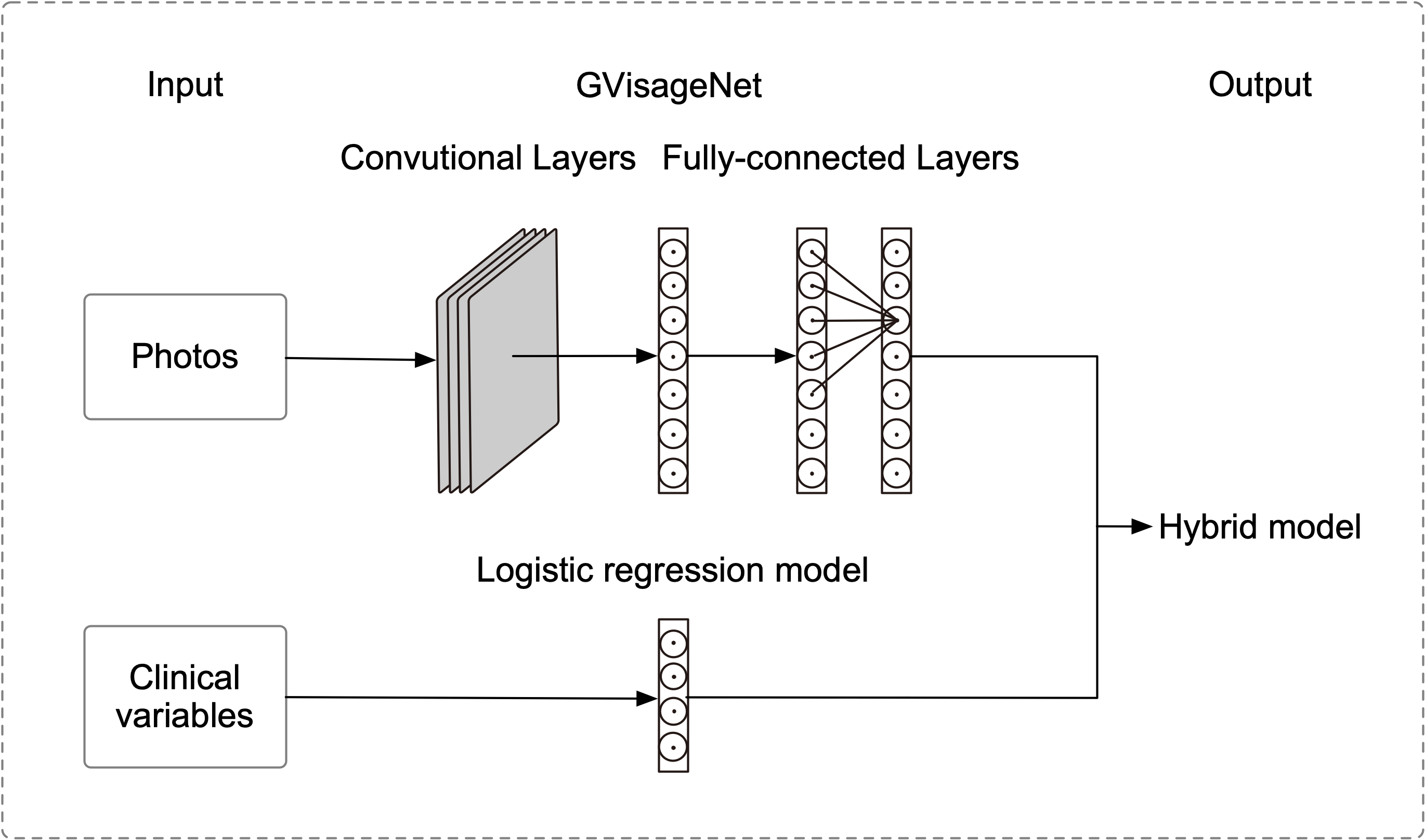
**

**Table S1.** The diagnosis of other midline brain tumors.

| **Variables, n** | **Training dataset** | **Validation dataset** | **Independent Test dataset** |
| --- | --- | --- | --- |
| Medulloblastoma | 32 | 7 | 7 |
| Pineal gland adenoma | 45 | 15 | 16 |
| Ependymoma | 9 | 8 | 2 |
| Pituitary adenoma | 11 | 3 | 2 |
| Optic nerve glioma | 78 | 18 | 14 |
| Menigioma | 14 | 8 | 4 |
| Metastatic tumor | 0 | 4 | 2 |
| Total | 189 | 63 | 47 |

**Table S2.** The 95% confidence interval of all diagnostic measurements.

| **Models** | **AUC** | **Accuracy** | **Sensitivity** | **S**pecificity |
| --- | --- | --- | --- | --- |
| **Validation Dataset (Max Sen+Spe)** ^*^ | | | | |
| DL Model | 0.705[0.590-0.760] | 71.71% [57.42%-77.11%] | 75.24% [70.89%-77.57%] | 53.40%[39.04%-66.10%] |
| Logistic Regression Model **^†^** | 0.512[0.213-0.569] | 56.41%[31.85%-74.28%] | 65.12%[50.78%-77.54%] | 42.93%[28.48%-65.74%] |
| Hybrid Model | 0.611 [0.676-0.758] | 63.77% [39.48%-77.65%] | 65.94% [48.99%-73.84%] | 58.78%[39.31%-72.53%] |
| **Test Dataset (Max Sen+Spe)** | | | | |
| DL Model | 0.739[0.688-0.793] | 70.00%[59.85%-75.34%] | 68.33%[36.65%-75.14%] | 63.24%[50.59%-80.00%] |
| Logistic Regression Model **^†^** | 0.572[0.471-0.649] | 62.97%[52.68%-75.44%] | 51.88%[47.45%-78.74] | 58.59%[49.24%-70.04%] |
| Hybrid Model | 0.669[0.398-0.723] | 66.56%[59.90%-73.84%] | 71.77%[56.48%-76.88%] | 68.45%[35.33%-77.05%] |

AUC denotes area under the receiver operating characteristic curve.

^†^ The logistic regression model included the following baseline variables: limb movement disorder, diabetes insipidus, sexual abnormality, affective disturbance, convulsive seizure, thyroid stimulating hormone, and growth hormone.

**References**

1. Derry A, Krzywinski M & Altman N. Convolutional neural networks. *Nat Methods* 2023 **20** 1269-1270.

2. Saharia C, Ho J, Chan W, Salimans T, Fleet DJ & Norouzi M. Image Super-Resolution via Iterative Refinement. *IEEE Trans Pattern Anal Mach Intell* 2023 **45** 4713-4726.

3. Lin CL & Wu KC. Development of revised ResNet-50 for diabetic retinopathy detection. *BMC Bioinformatics* 2023 **24** 157.

4. Sharma AK, Nandal A, Dhaka A, Koundal D, Bogatinoska DC & Alyami H. Enhanced Watershed Segmentation Algorithm-Based Modified ResNet50 Model for Brain Tumor Detection. *Biomed Res Int* 2022 **2022** 7348344.
